# Dissecting the mechanism of carotid atherosclerosis from the perspective of regulation

**DOI:** 10.3892/ijmm.2014.1960

**Published:** 2014-10-09

**Authors:** MIN LIN, LIN ZHAO, WENLONG ZHAO, JING WENG

**Affiliations:** 1 Department of Neurology, Fuzhou General Hospital of Nanjing Command, PLA, Fuzhou 350025, P.R. China; 2 Department of Neurosurgery, Fuzhou General Hospital of Nanjing Command, PLA, Fuzhou 350025, P.R. China

**Keywords:** carotid atherosclerosis, differentially expressed genes, transcription factors, miRNA

## Abstract

Carotid atherosclerosis is a chronic inflammatory disease of the arterial wall. The present study aimed to identify changes in the gene expression and regulatory factors for atherosclerotic plaques of carotid atherosclerosis from an early to an advanced stage. The original data were downloaded from the NCBI GEO database under accession no. GSE28829. Differentially expressed genes (DEGs) were detected by the Robust Multiarray Average (RMA). The enriched Gene Ontology (GO) terms and pathways for DEGs using DAVID were subsequently identified. The transcriptional and microRNA (miRNA) regulatory network were constructed for the DEGs. *Cis*-regulatory signals were also investigated. More genes were activated in the advanced stage compared with the early stage. IGHG1 and SPP1 were upregulated, while MYBL1 and PLD were downregulated. The upregulated genes in the advanced stage were involved in atherosclerosis-related GO terms such as immune, vascular and cell movement homeostasis. The DEGs were significantly enriched in cell adhesion molecules (CAMs) and the focal adhesion pathway. MMP9 and CFL2 played key roles in the transcriptional regulatory network. Moreover, miR-328 was identified as one of the hubs in the miRNA regulatory network. The results may therefore be used to determine the mechanism involved in carotid atherosclerosis.

## Introduction

Atherosclerosis is a chronic disease that remains asymptomatic for decades (1). It is caused by the formation of multiple plaques within the arteries. Attention has been focused on the ‘vulnerable plaque’ since the late 1990s onwards (2). Atherosclerosis can lead to ischemic heart disease, cerebrovascular accidents and peripheral vascular diseases (3). Carotid intima-media thickness (cIMT) level is known as a surrogate marker of atherosclerosis (4). A special type of carotid atherosclerosis with CagA-positive *Helicobacter pylori* (CagA^+^ HP) infection is common in China. An increased serum YKL-40 level suggests plaque instability and more severe clinical symptoms of carotid atherosclerosis with CagA^+^ HP infection (5). Inflammatory cytokines induced by VEGF, such as monocyte chemoattractant protein (MCP-1), have been previously shown to be involved in the pathogenesis and progression of carotid atherosclerosis (6).

Metabolic syndrome may be independently associated with the early stage but not the later and advanced stages of carotid atherosclerosis in community residents in China (7). An *in vivo* 3T MRI study was previously conducted to determine the effect of gender differences of high-risk carotid atherosclerotic plaque with <50% stenosis in asymptomatic patients (8). Evaluation of carotid atherosclerosis was therefore performed from the perspective of blood flow reflection (9). Results of a multivariate analysis revealed plaque number by ultrasonography (P=0.023), age (P=0.001), calcium-phosphate product (P=0.049) and serum albumin (P=0.009) as independent risk factors (10). Higher brachial-ankle pulse wave velocity was identified as a risk factor for carotid atherosclerosis in patients with end-stage renal disease (11). The relationship between levels of circulating intercellular cell-adhesion molecule-1 (cICAM-1) or P-selectin (cP-selectin) and the severity of carotid atherosclerosis was also examined (12). The findings of that study showed that cP-selectin did not increase until atherosclerosis was at an advanced stage (12).

Inactivation of the PDZK1 gene is known to promote the development of aortic root atherosclerosis in apolipoprotein E (apoE) KO mice fed with a high fat/high cholesterol diet (13). Additionally, SERPINA1 was found to be upregulated in atherosclerotic plaques (14). Angiogenesis, the process of new capillary formation from existing blood vessels, is dysregulated in many pathological disorders including atherosclerosis (15). In CD68^+^ cells from regressing plaque of atherosclerosis, Feig *et al* (16) observed that genes related to cell adhesion, such as cadherins and vinculin, were downregulated.

Transcription factors such as LDLR and TP53 are important in the development of atherosclerosis, as identified in a Malaysian study population (13). It has been previously reported that miRNAs are associated with atherosclerosis (17). However, the association between miRNAs and atherosclerosis has not yet been fully elucidated.

Immune-associated biological processes can affect atherosclerosis. Growth differentiation factor-15 deficiency inhibits the progression of atherosclerosis by controlling the interleukin-6-dependent inflammatory response to vascular injury (18). Vascular smooth muscle cell (VSMC) phenotypic modulation plays a key role in atherosclerosis. SMCs secrete cytokines and express cell adhesion molecules such as IL-8, IL-6 and VCAM-1 (19). Prenatal arsenic exposure alters gene expression in the adult liver to a proinflammatory state contributing to accelerated atherosclerosis by affecting pathways such as antigen processing and presentation (20). Vascular smooth muscle contraction is a main effect for artery (21). Morelloflavone, a biflavonoid and an active ingredient of the plant, has been shown to inhibit VSMC migration through its inhibition of multiple migration-related kinases such as focal adhesion kinase (22).

Therefore, in the present study, we initially detected differentially expressed genes (DEGs). Subsequently, we identified the enriched Gene Ontology (GO) terms and pathways for the DEGs. The transcriptional and miRNA regulatory network for the DEGs was also constructed. *Cis*-regulatory signals were also investigated.

## Materials and methods

### Data preprocessing

The original data were downloaded from the National Center for Biotechnology Gene Expression Omnibus (NCBI GEO) database for atherosclerotic plaques of carotid atherosclerosis under accession no. GSE28829 (23), including 13 chips at early stage (EAR, pathological, intimal thickening and intimal xanthoma) and 16 chips at advanced stage (ADV, thin or thick fibrous cap atheroma). The chip platform was GPL570, Affymetrix Human Genome U133 Plus 2.0 Array.

Background subtraction and quantile normalization was performed using Affymetrix Power Tools (APT) (http://www.affymetrix.com/) in the Robust Multiarray Average (RMA) algorithm (24). Genes with a low expression were filtered, ensuring that their plier was ≥100 in at least 2/3 of the samples (25).

### Identifying differentially expressed genes

Criteria for a differential probe included: i) signal strength of one probe is >1.5-fold between the two groups; ii) t-test P-value of ≤0.01; and iii) plier ≥100 in at least 2/3 of the samples.

### Functional enrichment of differential genes

Gene Ontology (GO) and the Kyoto Encyclopedia of Genes and Genomes (KEGG) pathway enrichment analysis was conducted with DAVID (26) for the differential genes between two groups. The threshold [P-value ≤0.05 and false discovery rate (FDR) ≤0.05] was calculated using Fisher’s Exact test.

### Transcriptional regulatory network

The differential genes, especially atherosclerosis-related genes, may be regulated by different transcriptional factors and miRNAs in different stages. We initally extracted the sequences located at 1k bp upstream and 200 bp downstream of 5′UTR for the transcription start site, using JASPAR (27) (http://jaspar.genereg.net) (the score threshold was 0.95). The core transcriptional regulatory network was constructed for the upregulated genes, with a >4-fold change in the advanced stage and a >2-fold change in the early stage. Upregulated genes were regulated by transcriptional factors (TFs) in the early and advanced stages of carotid atherosclerosis. TFs act depending on *cis*-regulatory motif in 5′UTR of their target genes. In order to detect such significant enriched cis-regulatory signals, hypergeometric distribution test was implemented (28), with a P-value of ≤0.05.

### miRNA regulatory network

The 1k bp 3′UTR sequence was extracted from the differentially expressed genes. Although the binding of miRNA and its targets in animals are not as conservative as in plant, while binding is relatively conservative in the seed region. RNAhybrid is based conservation in seed region, which makes it more suitable for animal miRNA target gene prediction. RNAhybrid (29) was subsequently used to identify miRNA binding sites (P-value ≤0.01).

## Results

### Differentially expressed genes

In the present study, the early stage sample was considered as the control group. We identified 889 differential probes (corresponding to 707 genes), of which 413 probes (322 genes) were downregulated, and 476 probes (385 genes) were upregulated in advanced stage samples.

**Figure 1:**
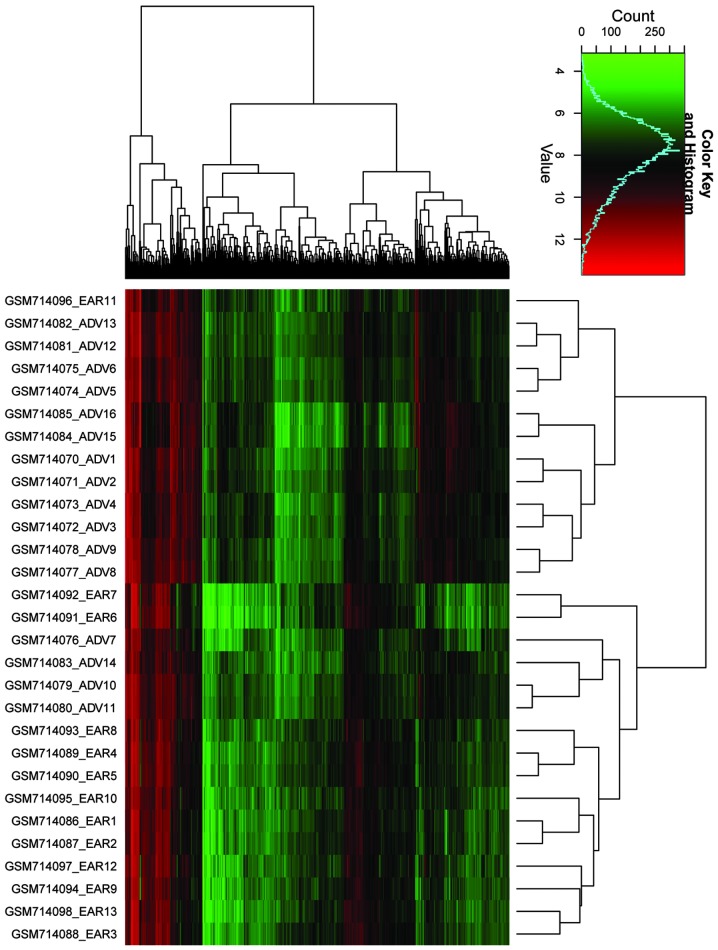
Hierarchical clustering analysis of 889 differentially expressed transcripts in all samples.

**Figure 2:**
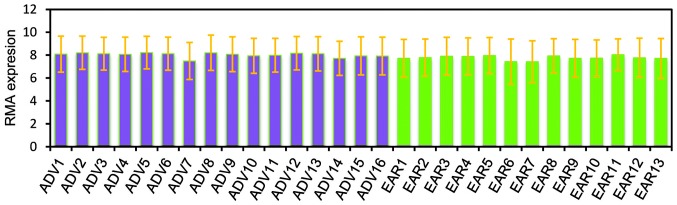
The average expression value of 889 differentially expressed transcripts in each sample. Purple bars are the advanced samples and green bars are the early stage sample. Bar values are the standard error of the mean (SEM).

**Figure 3:**
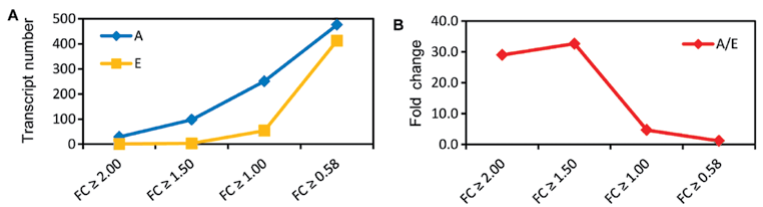
The distribution of 889 differentially expressed transcripts (DETs) in the early and advanced stages. (A) The number of transcripts with a different FC. Blue shows the advanced stage samples, while yellow shows the early stage samples. (B) The fold change of DETs in the advanced samples when compared with its expression in the early stage samples with a different FC. FC represents the log2 fold change.

The 889 differential probes were clustered using R hclust based on RMA log2-transformed values (Fig. 1). Thirteen samples were clustered into one group, with only one early stage sample being mis-clustered in this group. The standard error of the mean distribution is shown in Fig. 2. Fig. 3 shows the up- and downregulation for the 889 differential probes (corresponding to 708 genes). As the absolute log fold change (|logFC|) becomes larger, the number of upregulated genes were reduced (Fig. 3A). However, the fold change increased as the |logFC| became larger (Fig. 3B). For example, when |logFC|≥1.5, the fold change was 0.58, whereas when |logFC|≥1, the fold change was 1. This finding suggested that a large number of genes were activated in the advanced stage.

### Enriched functional terms of differential genes

The downregulated genes were enriched in biological processes including cytoskeleton organization, cell adhesion, muscle organ development, regulation of muscle contraction, regulation of cell growth, regulation of system process, regulation of metal ion transport, heart development, muscle contraction, muscle cell differentiation, negative regulation of cell growth, intracellular signaling cascade, myofibril assembly, cellular metal ion homeostasis, regulation of calcium ion transport, negative regulation of cell size and regulation of intracellular transport (Table I). The findings demonstrated that the biological processes do not correlate with atherosclerosis.

The upregulated genes enriched in biological processes associated with atherosclerosis were: i) immune-associated GO terms, including defense response, response to wounding, inflammatory response, immune effector process, leukocyte mediated immunity, antigen processing and presentation of peptide or polysaccharide antigen via the MHC class II, adaptive immune response, activation of immune response, lymphocyte mediated immunity, antigen processing and presentation, behavior, B cell-mediated immunity, antigen processing and presentation of exogenous peptide antigen, acute inflammatory response, chemotaxis, antigen processing and presentation of exogenous antigen; ii) vascular-related GO terms including blood vessel development, blood vessel morphogenesis, and angiogenesis; and iii) cell movement homeostasis including cell adhesion, regulation of cell motion, regulation of cell migration, regulation of locomotion, cation homeostasis and chemical homeostasis (Table II).

The enriched pathways of differential genes included systemic lupus erythematosus, antigen processing and presentation, complement and coagulation cascades, asthma, viral myocarditis, lysosome, intestinal immune network for IgA production (Table III).

### Transcriptional regulatory network

**Figure 4:**
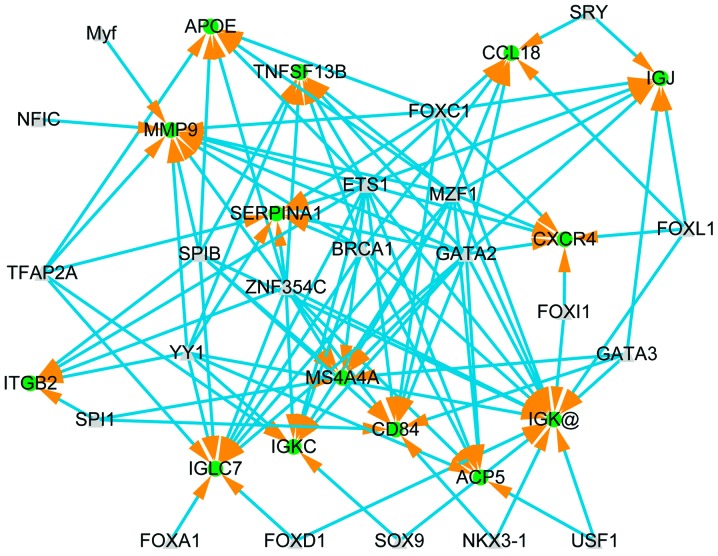
The core transcriptional regulatory network of upregulated genes (RMA log2 transformed values >2). Green nodes are the target genes, and the other nodes are the transcription factors. Arrows show that transcription factors target the genes.

The core transcriptional regulatory network is shown in Fig. 4 for upregulated genes (RMA log2 transformed values >2). Fourteen upregulated genes are shown, targeted by 21 transcription factors, constructing 111 regulatory relationships. The hub genes are IGK (15 neighbors), MMP9 (11 neighbors) and IGLC7 (10 neighbors).

**Figure 5:**
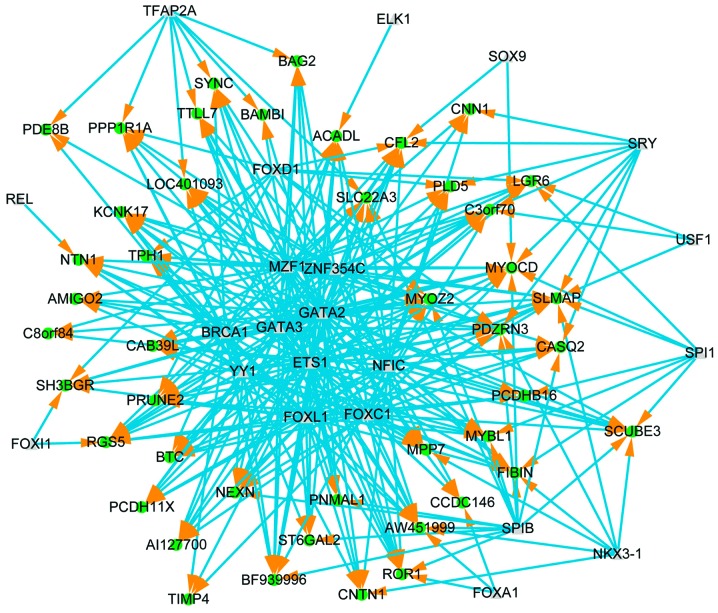
The core transcriptional regulatory network of downregulated genes (RMA log2 transformed values <-1). Green nodes are the target genes, and the other nodes are the transcription factors. Arrows show that transcription factors target the genes.

The core transcriptional regulatory network is shown in Fig. 5 for downregulated genes (RMA log2 transformed values <-1). Forty-five downregulated genes, targeted by 22 transcription factors, constructing 324 regulatory relationships. The hub genes are AW451999 (10 neighbors), CFL2 (10 neighbors) and PDZRN3 (10 neighbors).

### Significantly enriched cis-regulatory signals

**Figure 6:**
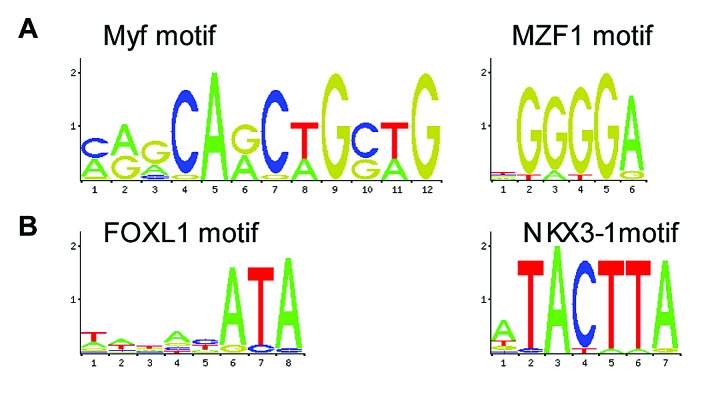
Significantly enriched TF motifs by DETs. Enriched motifs of (A) upregulated and (B) downregulated genes.

Fig. 6A shows the 5′UTR of upregulated genes enriched in TF motif Myf (P-value 1.33E-02) and MZF1 (P-value 2.47E-02). By contrast, Fig. 6B shows the 5′UTR of downregulated genes enriched in TF motif FOXL1 (P-value, 5.95E-03) and NKX3-1 (P-value, 3.57E-02).

### miRNA regulatory network

**Figure 7:**
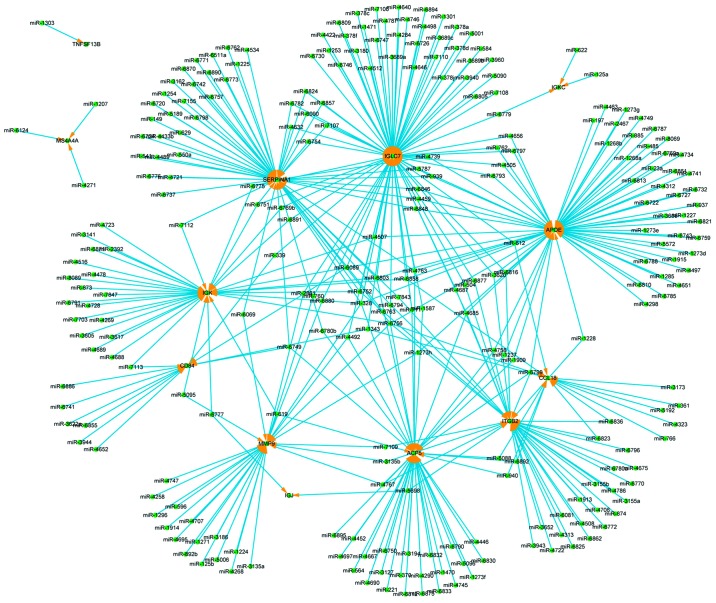
The core miRNA regulatory network of upregulated genes (RMA log2 transformed values >2) in advanced stage samples.

The core miRNA regulatory network is shown in Fig. 7 for the upregulated genes (RMA log2 transformed values >2). The genes identified by arrows are 13 upregulated genes, targeted by 262 miRNAs, comprising 372 regulatory relationships. The hub genes are IGLC7 (78 miRNAs target it), APOE (66 miRNAs) and SERPINA1 (53 miRNAs). The hub miRNAs are miR-6756 (8 target genes), miR-328 (6 genes) and miR-6803 (5 genes).

**Figure 8:**
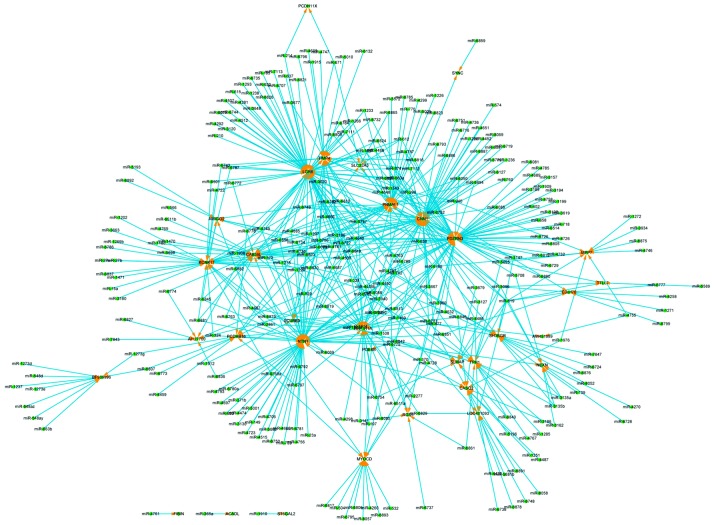
The core miRNA regulatory network of upregulated genes (RMA log2 transformed values >2) in the early stage samples.

The core miRNA regulatory network is shown in Fig. 8 for the downregulated genes (RMA log2 transformed values >1). The genes identified by arrows are 33 downregulated genes, targeted by 295 miRNAs, comprising 561 regulatory relationships. The hub genes are LGR6 (71 miRNAs target it), NTN1 (61 miRNAs), CNN1 (59 miRNAs) and PDZRN3 (57 miRNAs). The hub miRNAs are miR-6756 (11 target genes), miR-619 (10 genes), miR-6089 (8 genes) and miR-6803 (8 genes).

## Discussion

Carotid atherosclerosis is defined as the presence of atherosclerotic plaques in any of the carotid vessel segments (30). In the present study, we aimed to identify gene expression changes and regulatory factors for carotid atherosclerosis from an early to an advanced stage. More genes were activated in advanced stage compared with early stage. The upregulated genes in the advanced stage were involved in GO terms including immune, vascular and cell movement homeostasis. The differentially expressed genes (DEGs) were significantly enriched in cell adhesion molecules (CAMs) and focal adhesion. Genes such as MMP9 and CFL2 played key roles in the transcriptional regulatory network. Moreover, miR-328 was one of the hubs in the miRNA regulatory network.

A total of 889 transcripts were identified to be differentially expressed from early stage plaques of carotid atherosclerosis to advanced stage plaques. As shown in Fig. 3B, A/E increased when the fold change threshold was elevated. The majority of the DEGs were upregulated in the advanced stage, while they were inhibited in the early stage.

The DEGs activated in the advanced stage may correlate with plaques of carotid atherosclerosis and various types of cancer. A number of immune system-related cells were detected in human carotid atherosclerosis patients such as monocytes/macrophages, T cells and plasmacytoid dendritic cells (pDCs) (23). IGHG1 (Ig γ-1 chain C region) is one gene associated with ‘innate immune response’. IGHG1 expression has been reported to correlate with immune evasion mechanisms, which contribute to the proliferation of human pancreatic cancer (31). Furthermore, inhibiting IGHG1 expression by siRNA leads to cancer growth inhibition and apoptosis in prostate cancer (32). SPP1 (osteopontin) is involved as a cytokine in type I immunity to elevate the product of interferon-γ and interleukin-12. On the other hand, SPP1 reduces the expression of interleukin-10, and IFN-γ treatment, resulting in an increase of cytokines/cytokine receptors including CSF2, IL1R2 and SPP1 (33). In addition, smoking can increase SPP1 expression, and subsequently induce inflammation and emphysema (34). IGKC (Ig κ chain C region) participates in humoral immune response, which has been reported to play key roles in non-small cell lung cancer and breast cancer (35–37). The genes activated in early stage do not possess a similar function, including BTC, MYBL1 and PLD.

Immune-associated GO terms, vascular-related GO terms and cell movement homeostasis were identified to be associated with atherosclerosis. The IL-6-gp130 axis is a key regulator of inflammatory acute phase signaling in hepatocytes for the development of atherosclerosis (38). Blood flow is crucial for blood vessel development during embryogenesis and for the regulation of vessel diameter in adult life. It is also a key factor in atherosclerosis, which occurs mainly in regions of arteries that experience disturbances in fluid flow (39). Fibrinolytic balance and the potential contribution of PAI-1 to the regulation of cell migration are involved in the pathogenesis of the simple atherosclerotic lesions observed in the mouse (40).

Leukocyte transendothelial migration is one of the earliest events of immune inflammatory responses and may contribute to atherosclerosis (41). Immunologic arterial injury due to allograft rejection acting in synergy with hypercholesterolemia resulting from a dietary supplement of cholesterol can lead to rapidly developing atherosclerosis (42). Autoimmune thyroid disease has a causal relationship with atherosclerosis (even if mediated through traditional risk factors) (43). Focal adhesion plays key roles in VSMCs. Focal adhesion pathways may be expected to facilitate the formation of atherosclerotic plaques in ApoE-null mice (44). Systemic lupus erythematosus (SLE) is a systemic autoimmune disease that is characterized by autoantibody production and inflammatory disease involving multiple organs. Premature atherosclerosis is a common complication of SLE and results in substantial morbidity and mortality from cardiovascular disease (CVD) (45). Diabetes and atherosclerosis are associated with disorders of lipids and lipoproteins, notably high apolipoprotein B (apoB) and low apolipoprotein A1 (apoA1) are well established (46). Type I diabetes mellitus was also enriched by differential genes in this study. The passage of leukocytes across the endothelium and into arterial walls is a critical step in the development of atherosclerosis. It is consistent with our observation that DEGs were enriched in the pathway ‘leukocyte transendothelial migration’ (47).

The expression levels of matrix metallopeptidase 9 (MMP9) were assessed. MMP9 was potentially important in the development of atherosclerosis in a Malaysian study population (13). Cystic fibrosis transmembrane conductance regulator (CFTR) has a similar function to ABCA1. Schmitz and Buechler (48) identified ABCA1 as the major regulator of plasma high density lipoprotein (HDL) cholesterol. HDL metabolism is crucial in the prevention of the progression of atherosclerosis. CFL2 is an interactor of CFTR as reported by Wang and colleagues (49). An *in vivo* ApoE^−/−^ mouse model was utilized to assess the effects of chronic moderate exposure to arsenic on plaque formation and composition in order to facilitate mechanistic investigations (50). Arsenic exposure increases oxidative stress, inflammation and atherosclerotic lesion formation in ApoE^−/−^ mice (51). In human arterial tissue, SERPINA1 was upregulated (6.3-fold) in atherosclerotic plaques (14). In the present study, SERPINA1 was also upregulated in the advanced stage.

In this study, we also found that miR-328 may be crucial for atherosclerosis. miR-328 was linked to multiple upregulated genes in advanced stage samples and miR-328 has been found to be antiangiogenic (52). Anti-angiogenic perfluorocarbon nanoparticles has already used for diagnosis and treatment of atherosclerosis (53). Recently, in the ABCG2-positive cancer cells, miR-328 has been reported to regulate the expression of BCRP/ABCG2 (54). Additionally, miR-328 expression in plasma was significantly increased in atrial fibrillation (AF) patients (55).

Motifs enriched by upregulated genes in the early and advanced stages including Myf, MZF1, FOXL1 and NKX3-1, which were not investigated extensively were also investigated. These transcriptional factors may play pivotal roles in carotid atherosclerosis. In the present study, we identified gene expression changes and regulatory factors in carotid atherosclerosis. These results may facilitate in identifying the mechanism involved in carotid atherosclerosis.

## Figures and Tables

**Table I:** Major biological processes enriched by upregulated genes in early stage.

Term	Count	P-value	FDR
GO:0007010: Cytoskeleton organization	25	2.12E-07	3.51E-04
GO:0007155: Cell adhesion	29	1.14E-05	1.88E-02
GO:0007517: Muscle organ development	14	4.57E-05	7.56E-02
GO:0006937: Regulation of muscle contraction	7	1.16E-03	1.90E+00
GO:0001558: Regulation of cell growth	11	1.38E-03	2.25E+00
GO:0044057: Regulation of system process	14	1.80E-03	2.93E+00
GO:0010959: Regulation of metal ion transport	7	2.13E-03	3.46E+00
GO:0007507: Heart development	11	2.92E-03	4.73E+00

P≤0.05 and false discovery rate (FDR) ≤0.05.

**Table II:** Major biological processes enriched by upregulated genes in advanced stage.

Term	Count	P-value	FDR
GO:0006955: Immune response	86	4.85E-40	8.42E-37
GO:0006952: Defense response	65	1.40E-25	2.43E-22
GO:0009611: Response to wounding	57	1.10E-22	1.91E-19
GO:0006954: Inflammatory response	43	2.13E-20	3.69E-17
GO:0006935: Chemotaxis	27	2.20E-15	3.85E-12
GO:0042330: Taxis	27	2.20E-15	3.85E-12
GO:0002252: Immune effector process	21	2.08E-11	3.61E-08
GO:0048584: Positive regulation of response to stimulus	27	2.37E-11	4.11E-08
GO:0007626: Locomotory behavior	29	2.40E-11	4.16E-08
GO:0002443: Leukocyte mediated immunity	17	7.37E-11	1.28E-07
GO:0002504: Antigen processing and presentation of peptide or polysaccharide antigen via MHC class II	12	8.24E-11	1.43E-07
GO:0002250: Adaptive immune response	16	1.47E-10	2.55E-07
GO:0002253: Activation of immune response	17	2.97E-10	5.16E-07
GO:0002449: Lymphocyte mediated immunity	15	4.22E-10	7.33E-07
GO:0019882: Antigen processing and presentation	16	4.49E-10	7.79E-07
GO:0007610: Behavior	33	2.54E-08	4.41E-05
GO:0019724: B cell-mediated immunity	12	3.99E-08	6.92E-05
GO:0002478: Antigen processing and presentation of exogenous peptide antigen	7	5.36E-08	9.29E-05
GO:0007155: Cell adhesion	40	1.72E-07	2.98E-04
GO:0001568: Blood vessel development	22	1.75E-07	3.04E-04
GO:0002526: Acute inflammatory response	14	3.02E-07	5.23E-04
GO:0019884: Antigen processing and presentation of exogenous antigen	7	3.29E-07	5.70E-04
GO:0001775: Cell activation	23	6.13E-07	1.06E-03
GO:0030334: Regulation of cell migration	17	1.43E-06	2.48E-03
GO:0048514: Blood vessel morphogenesis	19	1.45E-06	2.51E-03
GO:0051270: Regulation of cell motion	18	1.83E-06	3.18E-03
GO:0055066: Di-, tri-valent inorganic cation homeostasis	20	2.13E-06	3.69E-03
GO:0045321: Leukocyte activation	20	2.56E-06	4.44E-03
GO:0006956: Complement activation	9	3.78E-06	6.56E-03
GO:0002541: Activation of plasma proteins involved in acute inflammatory response	9	4.55E-06	7.90E-03
GO:0001525: Angiogenesis	15	6.60E-06	1.14E-02
GO:0042592: Homeostatic process	38	6.64E-06	1.15E-02
GO:0040012: Regulation of locomotion	17	7.55E-06	1.31E-02
GO:0009617: Response to bacterium	17	8.07E-06	1.40E-02
GO:0055080: Cation homeostasis	21	8.17E-06	1.42E-02
GO:0050865: Regulation of cell activation	16	1.04E-05	1.80E-02
GO:0048878: Chemical homeostasis	29	1.48E-05	2.57E-02
GO:0006874: Cellular calcium ion homeostasis	16	1.77E-05	3.07E-02

P≤0.05 and false discovery rate (FDR) ≤0.05.

**Table III:** Enriched pathways of differentially expressed transcripts.

Term	Count	P-value	FDR
hsa05322: Systemic lupus erythematosus	21	3.65E-08	0.00004
hsa04514: Cell adhesion molecules (CAMs)	23	2.78E-07	0.00033
hsa04612: Antigen processing and presentation	18	3.26E-07	0.00039
hsa04610: Complement and coagulation cascades	16	7.32E-07	0.00087
hsa05310: Asthma	10	5.73E-06	0.00681
hsa05416: Viral myocarditis	15	5.92E-06	0.00704
hsa04142: Lysosome	19	1.13E-05	0.01347
hsa04672: Intestinal immune network for IgA production	12	1.66E-05	0.01978

P-value ≤0.05 and false discovery rate (FDR) ≤0.05.
